# Supplementary material for: Hyperferritinemia Screening to Aid Identification and Differentiation of Patients with Hyperinflammatory Disorders
Source: J Clin Immunol. 2024 Sep 12;45(1):4. doi: 10.1007/s10875-024-01797-4 (PMC11393296; doi:10.1007/s10875-024-01797-4)
Supplement: Supplementary file 1 — Supplementary file1 (PDF 1.21 MB) [file 10875_2024_1797_MOESM1_ESM.pdf]

	Events			Patients		
**total**	**931**			**180**		
**Inflammatory**	**375**			**55**		
Rheumatic*		105			8	
Infection		179			30	
Epstein-Barr Virus			20			5
Adenovirus			17			2
Sepsis[a]			79			12
Other[a]			63			11
Immune Dysregulation[b]		91			17	
Hepatitis-associated			12			8
**Anemia**	**366**			**48**		
Sickle Cell		266			30	
Thalassemia		35			4	
Other[1]		65			14	
**Transplant**	**149**			**56**		
HSCT		113			41	
Cardiac		12			6	
Renal		8			4	
Mutliple		5			2	
Other[2]		11			3	
**Oncology**	**37**			**17**		
Leukemia		17			8	
Neuroblastoma		9			4	
Lymphoma		9			3	
Other[3]		2			2	
**Not otherwise classified[4]**	**4**			**4**		

*All were systemic Juvenile Idiopathic Arthritis (JIA) or Adult-onset Still's Disease

a-Underlying conditions in septic patients included William Syndrome, prematurity, Prune Belly Syndrome, short gut syndrome. "other" diagnoses include cystic fibrosis pneumonia, influenza, malaria.

b-17p triplication, XIAP deficiency, Langerhans Cell Histiocytosis, Trisomy 18, sulfa hypersensitivity, Immuno-osseous dysplasia

1-blackfan diamond anemia (2), anemia secondary to hemorrhage, congenital dyserythropoeitic, chronic renal disease, Pearson Syndrome, hemolytic anemia (2), and Arthrogryposis-renal dysfunction-cholestasis syndrome.

2-liver, lung

3-wilm's, rhabdomyosarcoma

4-GM2 synthase deficiency, cri-du-chat syndrome, Neonatal hemochromatosis, myasthenia gravis/hashimoto's thyroidtitis

Table S1. Specific diagnoses per umbrella disease group. Number of alerts and distinct patients per individual diagnosis are displayed.

Diagnosis	Number of patients identified
MIS-C or active COVID-19, IHF	24
SJIA/MAS, IHF	3
MAS (not secondary to SJIA), IHF	2
HLH, IHF	4
Kawasaki disease, IHF	1
Meningococcal sepsis, IHF	1
Leukemia/lymphoma	5
Anti-RBC antibody	1
Ingestion	2
Sickle Cell Disease	4
Myelodysplastic syndrome	1
Epidermolysis bullosa	1
Solid Organ Transplant	3
B-thalassemia	5
Neuroblastoma	2
Fanconi anemia	1
Sarcoma	1
Aplastic Anemia	2
chronic transfusion NOS	1
Hepatoblastoma	1
Congenital heart	1
Langerhans cell histiocytosis	1
End-stage renal disease	2
Hemophilia	1
Unknown	5
	Total: 75

Table S3: Specific diagnosis and number of patients identified in CCHMC 6 month hyperferritinemia screen.
Abbreviations: NOS= not otherwise specified

Groups	n
HC	10
SJIA-MAS	18
HLH-genetic	5
HLH-malignancy	5
HLH-infection	8
Sepsis	25
Sepsis-bacterial	*13*
Sepsis-viral	*8*
Sepsis-bacterial+viral	*4*
CRS grade 0	6
CRS grade 1	9

Table S4. Hyperferritinemic sample breakdown

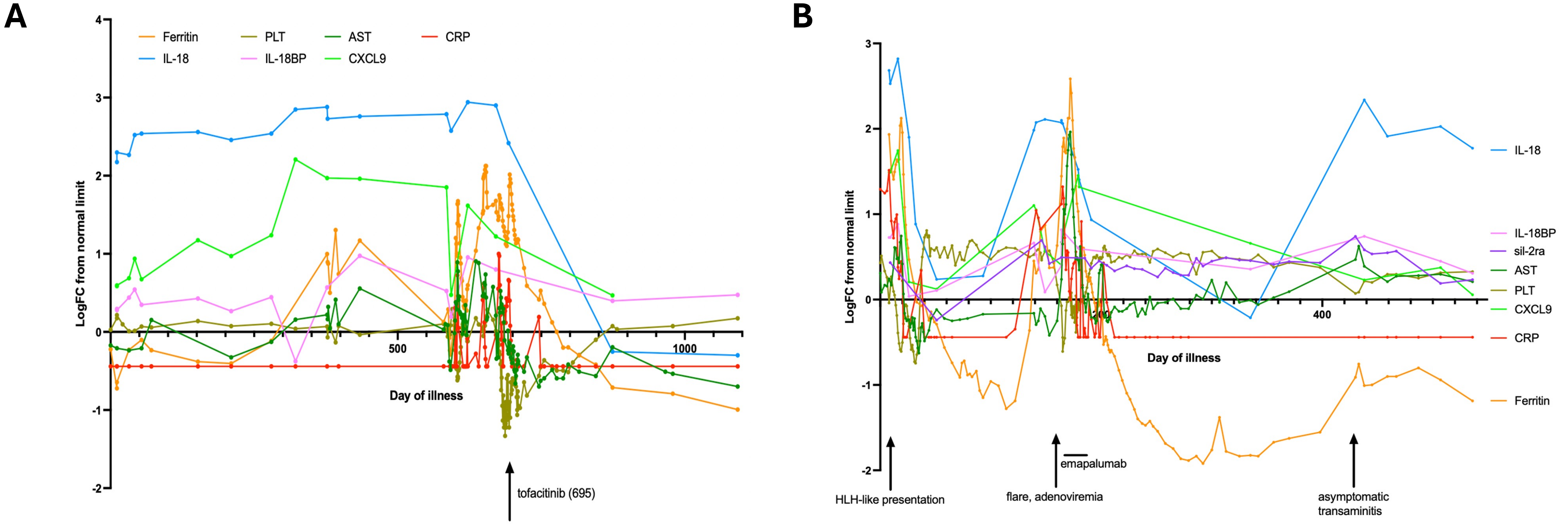

Figure S1. Longitudinal biomarkers of HLH/MAS. Key laboratory features throughout this patient's hospital course. The y-axis represents the Log10 fold change from the upper limit of normal (ferritin 500ng/mL, AST 55U/mL, CRP 0.8mg/dL, IL-18 540pg/mL, and CXCL9 1,000pg/mL), median of healthy controls (used because the range of normal is not well established for IL-18BP 5,000pg/mL), or lower limit of normal (platelet count PLT $150x10^9$/L). A. <u>Cases presentation</u>: An 18 year old female presented with fevers, rash, hyperferritinemia and arthritis and was diagnosed with AOSD. She was initially on prednisone and anakinra therapy, but her course was characterized by long-standing arthritis and compliance challenges. Over time, she developed prolonged hyperferritinemia. Due to intermittent MAS features, she was treated with corticosteroids, various IL-1 antagonists, IL-6 blockade, and cyclosporine. Eventually, she was admitted around day of illness 650 (from study enrollment) due to a seemingly untriggered disease flare. Her MAS-like symptoms worsened and she developed transient pulmonary hypertension and heart strain. She was treated with weekly pulse-dose methylprednisolone and tofacitinib at a maximum of 20mg twice daily. She showed robust improvement. Retrospective evaluation of IL-18 and related biomarkers showed persistent IL-18 elevation throughout her course until initiation of pulse methylprednisolone and tofacitinib. Her IL-18BP levels throughout this period were rarely elevated, but CXCL9 levels were persistently high in the period preceding her flare/admission. <u>B. Cases presentation</u>: A 4 month old female with Trisomy 21 presented with prolonged fever and a non-descript erythematous rash with labs suggesting an HLH-like presentation. She was initially treated with corticosteroids and completed 8 weeks' induction with the HLH-94 protocol (36) with remission of symptoms. Whole exome sequencing was notable for a heterozygous *PRF1* p.Gly220Ser mutation previously associated with fHLH in homozygosity or compound heterozygosity (37). She had normal perforin protein expression and normal NK cell function. Retrospectively, she was noted to have had extraordinarily high total IL-18 levels with near-normalization during remission. Several months later, she returned with fevers, but without other features for HLH and was found to have adenovirus PCR positivity. Subsequently she developed fulminant HLH and received methylprednisolone, high-dose anakinra, and ultimately three doses of emapalumab (a monoclonal antibody targeting IFNγ). She responded well to therapy, clearing adenovirus, and had normalization of her IL-18. Later, despite clinical remission, her transaminases became elevated, and her total IL-18 became significantly elevated, concerning for subclinical disease activity. She has been treated with chronic tofacitinib therapy and has not had a recurrence of fulminant HLH.

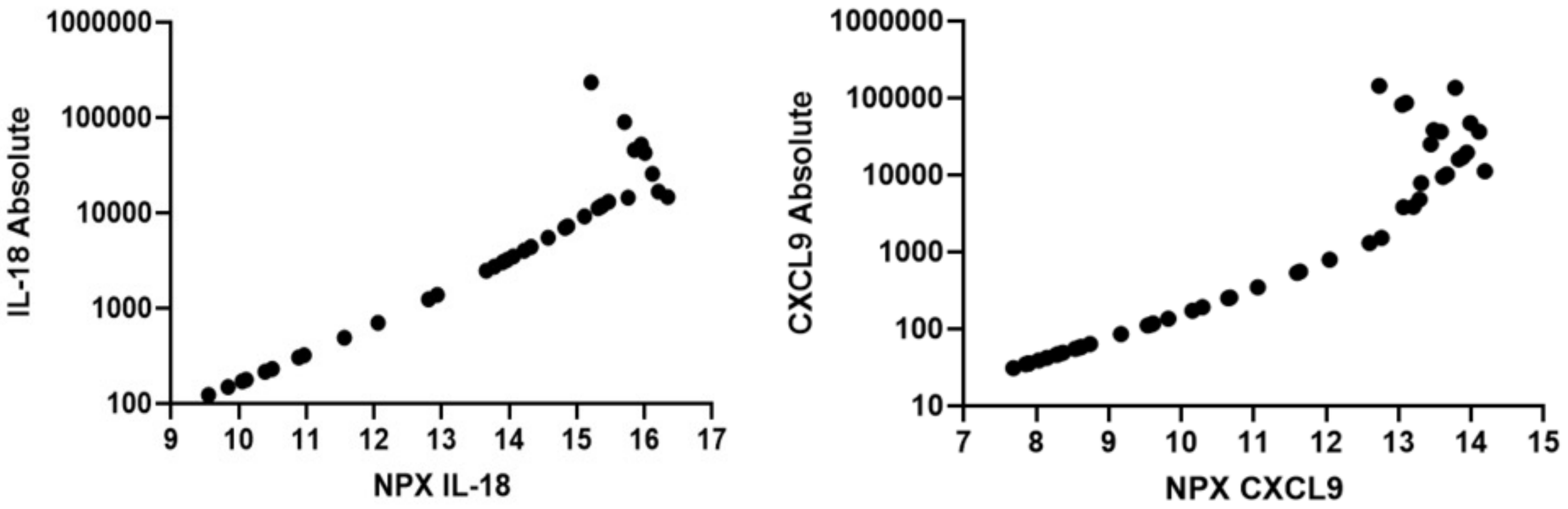

Figure S2. Correlation plots between O-link NPX values and absolute values in a data set of heathy control, patients with systemic JIA in remission, active systemic JIA, and systemic JIA in MAS. Both IL-18 and CXCL9 plots show the hook effect.

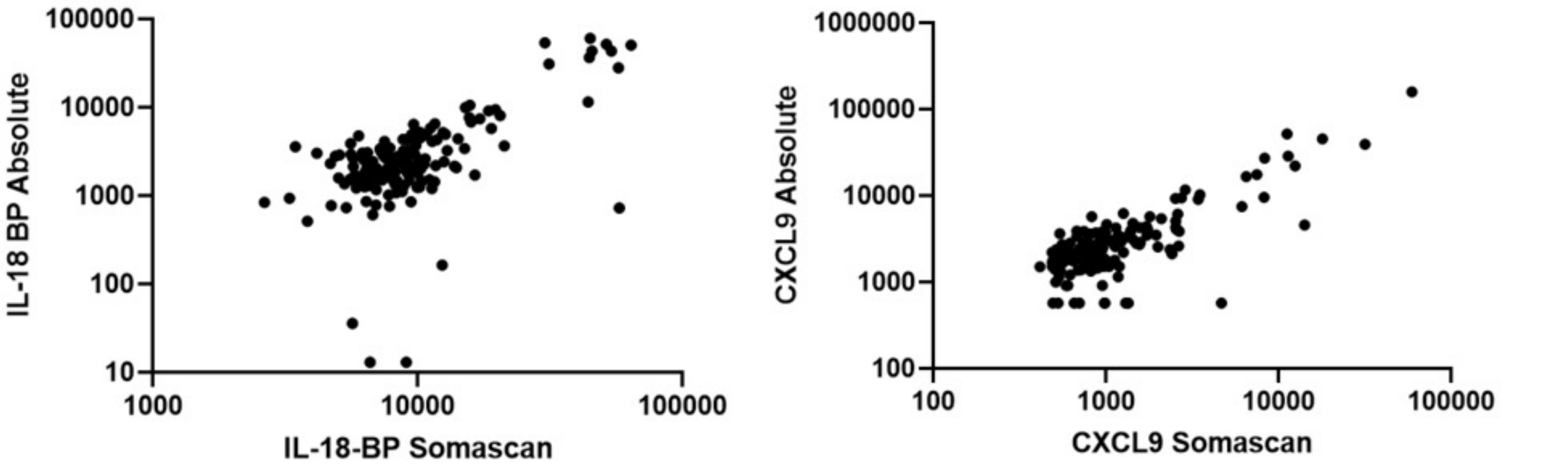

Figure S3. Correlation plots between Somascan and absolute values in a data set of heathy control, patients with inactive systemic JIA, active systemic JIA, systemic JIA in MAS, systemic JIA with pulmonary alveolar proteinosis (PAP), systemic JIA with PJP colonization, active neonatal-onset multisystem inflammatory disease (NOMID), and active STING-associated vasculopathy with onset in infancy (SAVI). Both IL-18 and CXCL9 plots do not show the hook effect.

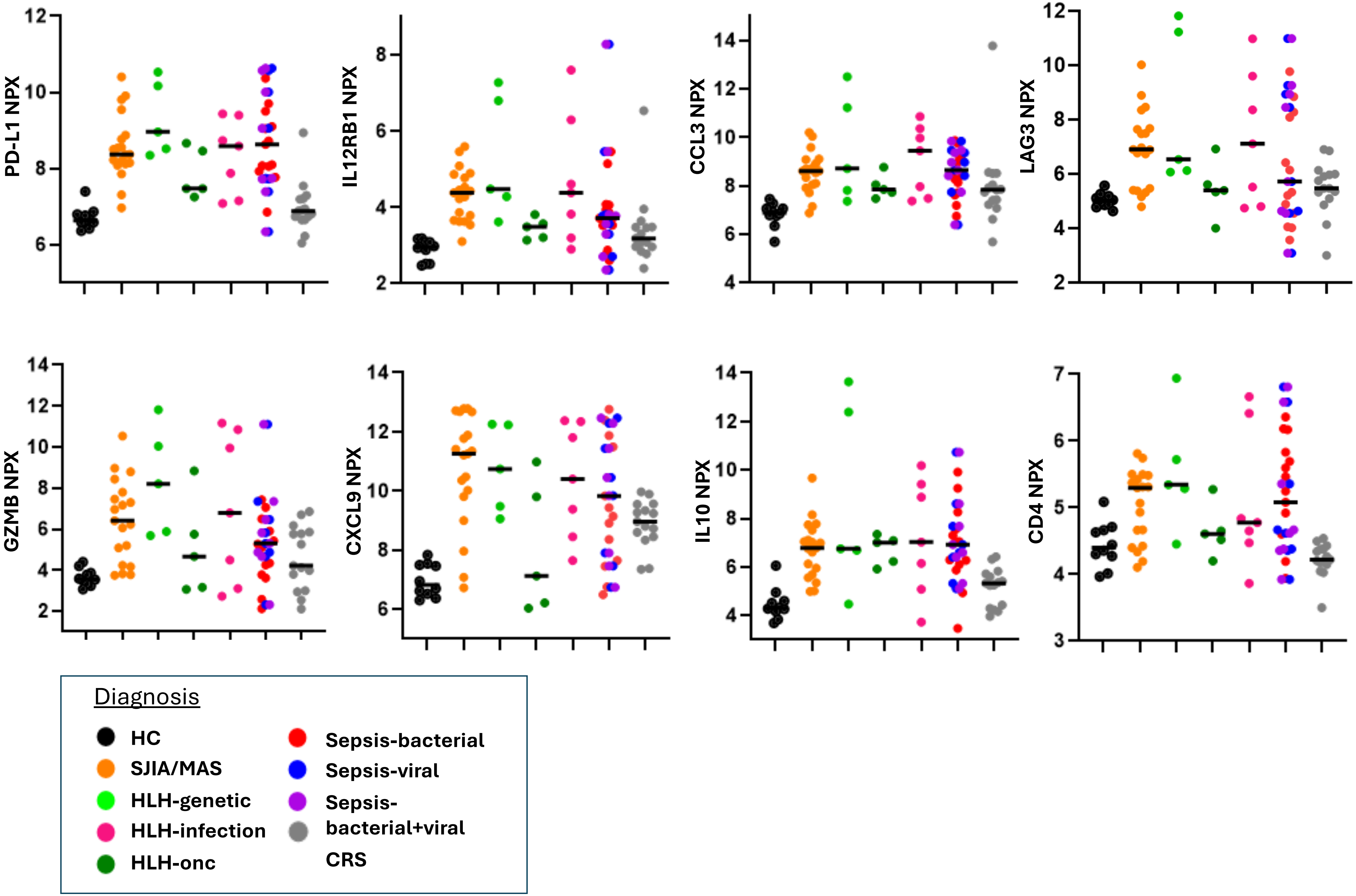

Figure S4. Top 8 analytes contributing to PC1.

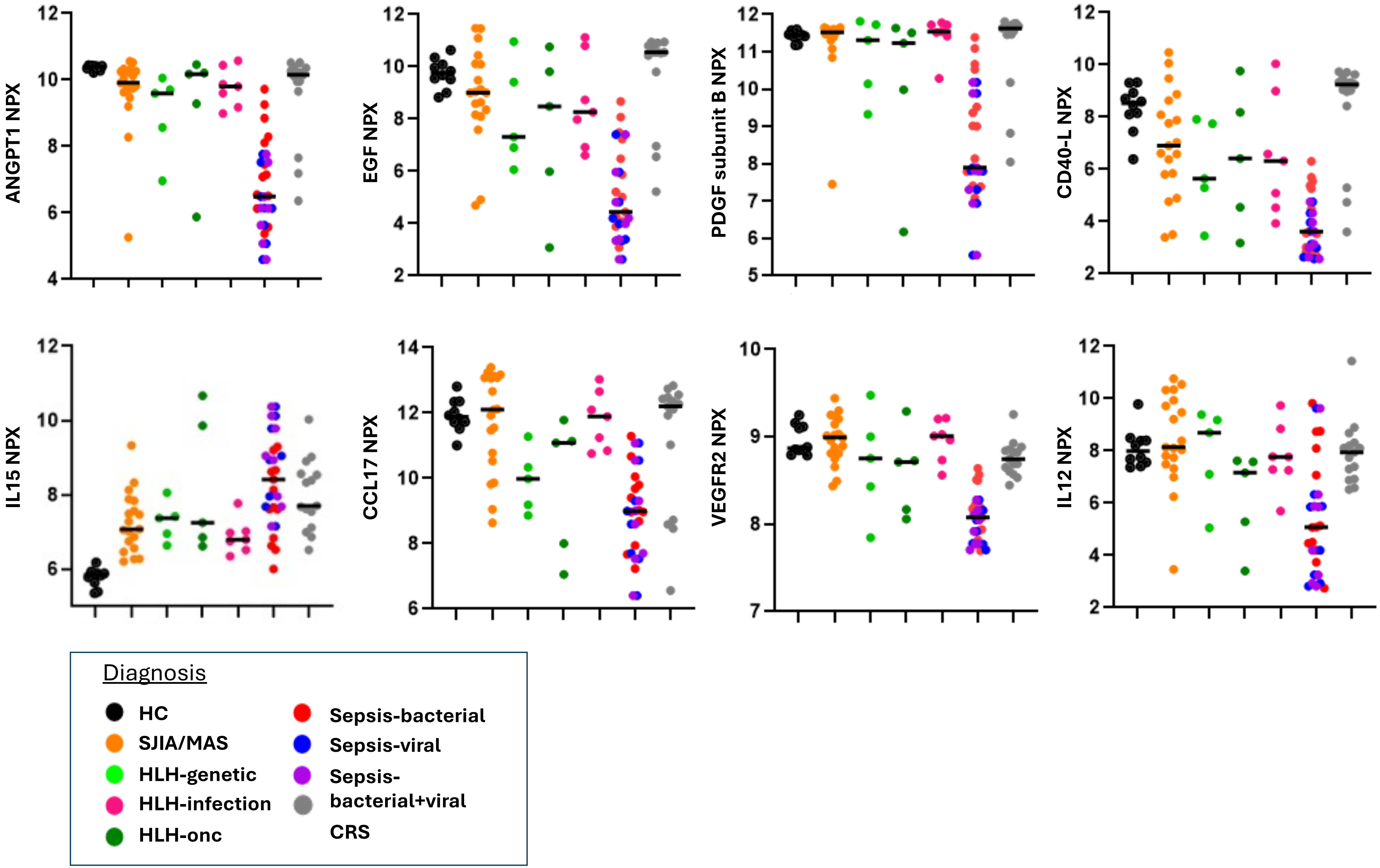

Figure S5. Top 8 analytes contributing to PC2.

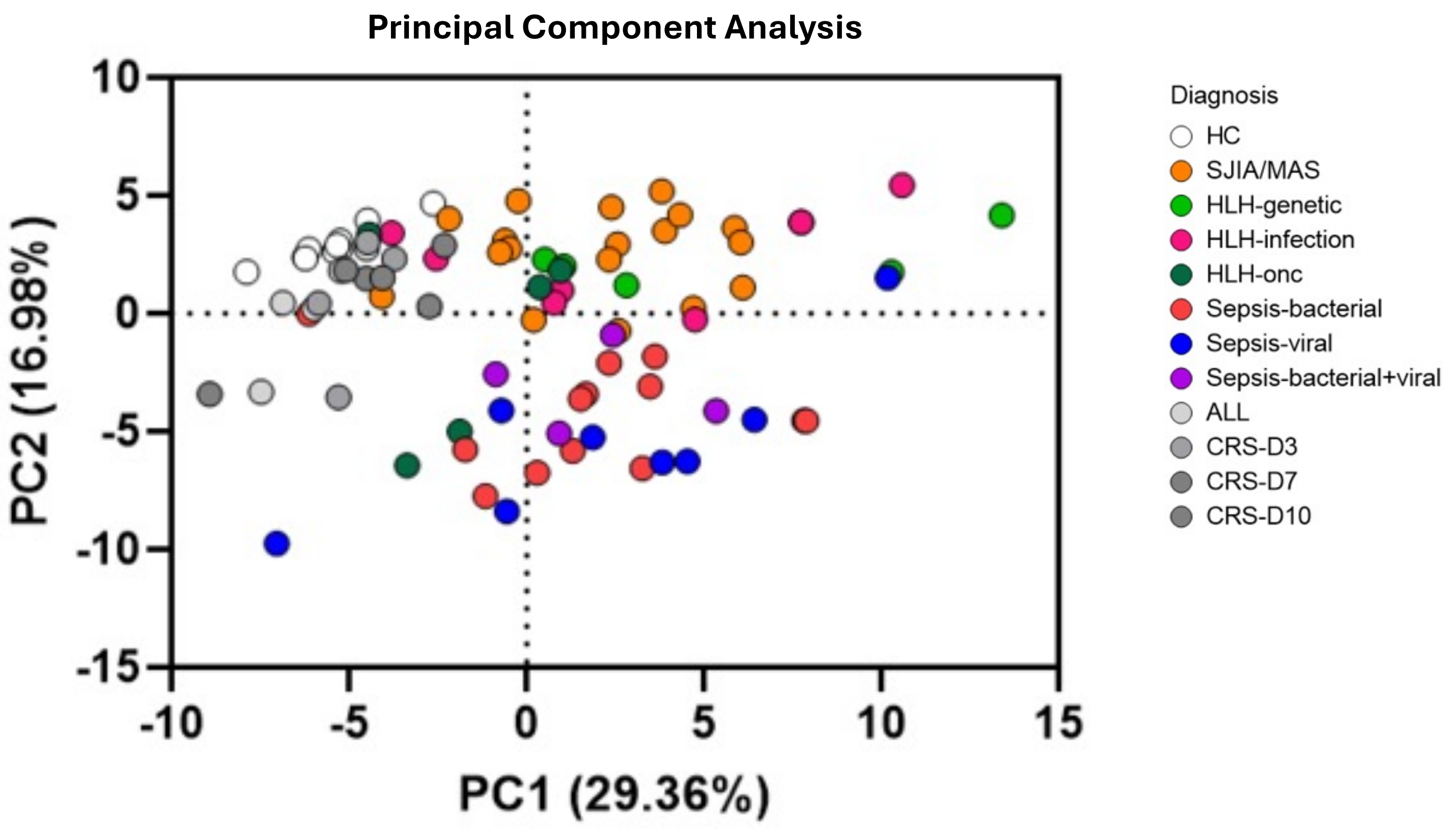

Figure S6. Principal Component Analysis of hyperferritinemic patients including the CRS cohort that has a range of ferritins ranging from 35-3000 ng/ml .

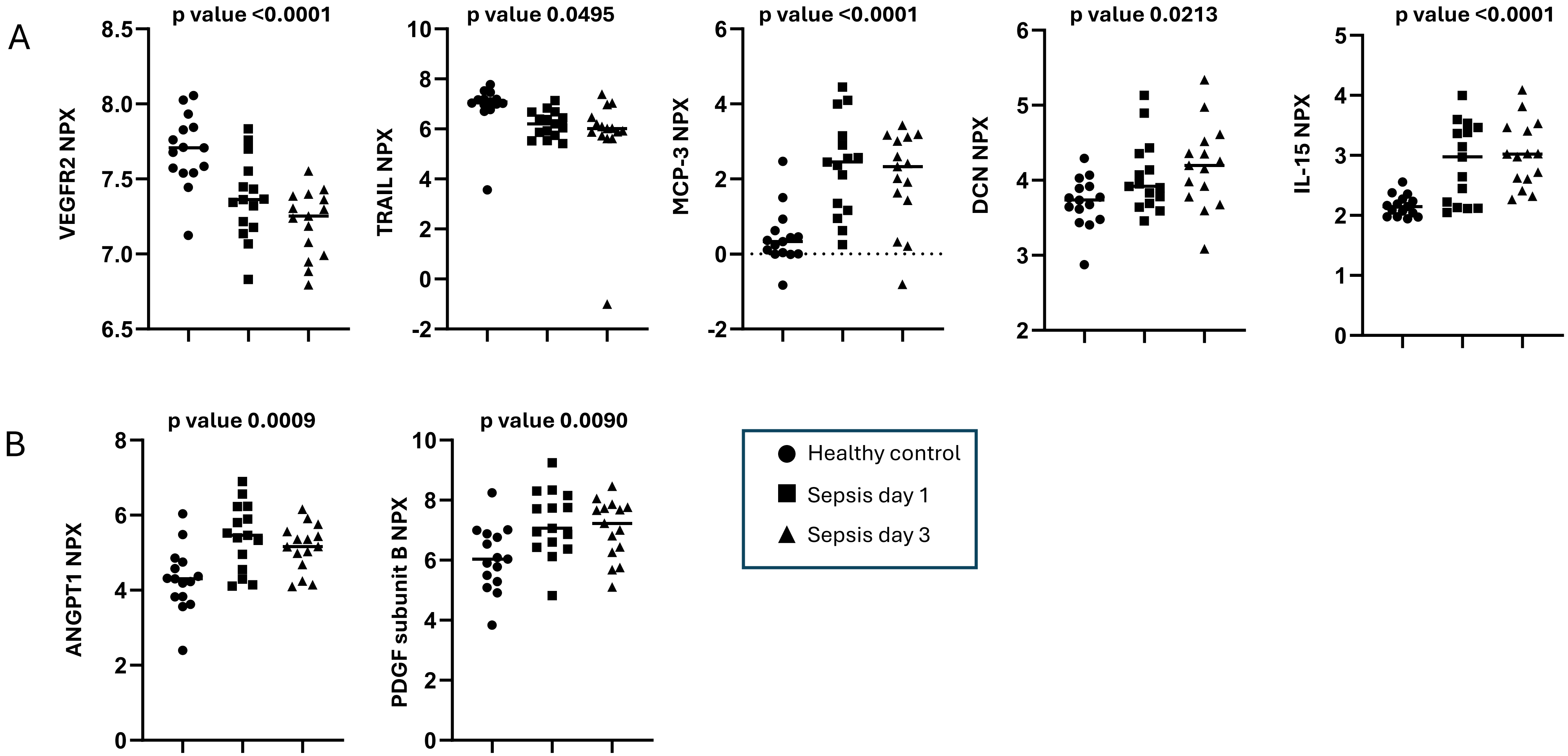

Supplemental Figure 7. The top 12 analytes identified in PC2 were analyzed in Van Nynatten et al data set to see general trends between (16). A. When compared to healthy controls, analytes with ANOVA p-value <0.05 and with the sepsis samples trending in the same direction as PC2 are displayed. B. When compared to healthy controls, analytes with ANOVA p-value <0.05 and opposite trending in the same direction as PC2 are displayed.
